# Hypoxia-Induced Downregulation of miR-29 in Renal Tumor Cells Affects Collagen IV Subunit Expression through Multiple Sites

**DOI:** 10.3390/biomedicines10123286

**Published:** 2022-12-19

**Authors:** Chuncheng Liu, Linan Liu, Jinlai Bo, Xian Lu, Donghui Qu, Gehui Liu, Zhiyan Jiang, Lu Cai

**Affiliations:** 1 School of Life Science and Technology, Inner Mongolia University of Science and Technology, Baotou 014010, China; 2 Inner Mongolia Key Laboratory of Functional Genome Bioinformatics, Inner Mongolia University of Science and Technology, Baotou 014010, China

**Keywords:** miR-29, collagen IV, extracellular matrix, 3’UTR, hypoxia

## Abstract

Multiple tumor exacerbations and treatment procedures, such as extracellular matrix remodeling, metabolic reprogramming, immunological evasion, and resistance to chemotherapy and radiotherapy, are influenced by intratumoral hypoxia. It is becoming increasingly clear how hypoxia interacts with the extracellular matrix and how this affects the growth of cancer. We analyzed the published sequencing results of hypoxia-stressed mouse kidney tumor cells and found that the expression of miR-29b was significantly downregulated. There are several sites that are complementary to the miR-29 seed sequence in the 3’ non-coding regions (3’UTRs) of various extracellular matrix-related genes, including collagen IV. We analyzed the sequences of the 3’UTRs of different subunits of collagen IV in different species and constructed the corresponding phylogenetic trees. We found that the 3’UTRs of *Col4a1* and *Col4a4* may have been subjected to particular evolutionary pressures. By cloning the 3’UTRs of collagen IV subunits into the psiCHECK^TM^-2 vector, we found that seven of the eight sites in the *Col4a3*–*Col4a6* gene complementary to miR-29 were significantly repressed by miR-29a, b (except for the 7774–7781 of *Col4a3* gene). The inhibitory efficiency of miR-29a, b on these seven sites was between 27% and 57%. The research on the regulation of miR-29 and extracellular matrix by hypoxia can provide a theoretical basis for tumor and fibrosis research and treatment.

## 1. Introduction

Renal cell carcinoma is a common solid tumor of the kidney. One-third of renal cell carcinomas will metastasize, and nearly half of the patients will die of this disease directly [1,2]. Renal cell carcinoma can be divided into three subtypes, of which 70–75% are clear cell renal cell carcinomas (ccRCC) [3]. ccRCC is closely associated with VHL (Von Hippel Lindau) protein inactivation [4]. Inactivation of VHL protein leads to abnormal accumulation of the transcription factors HIF1A and HIF2A, resulting in altered cellular metabolic pathways [5]. For the other two types of renal cell carcinomas (papillary RCC and chromophobe RCC), although they are not associated with VHL protein inactivation, HIF1A activation will also occur [6,7]. Alterations in metabolic pathways caused by HIF1A are important for the initiation and progression of renal cell carcinoma [8].

The extracellular matrix (ECM) is a key component of the tumor microenvironment that similarly influences tumorigenesis and metastasis. Hypoxia and alterations in cellular metabolic pathways can affect ECM deposition, remodeling, and degradation [9]. MiRNAs play an important role in the tumor hypoxia response. Therefore, hypoxia may affect the extracellular matrix of tumor cells by regulating the expression of miRNAs. 

MiR-29 is an anti-fibrotic miRNA; its expression changes are associated with fibrosis of various tissues and organs such as the heart, lungs, liver, skin, and kidneys [10]. For the kidney, the inhibition of miR-29 is closely related to the deposition of collagen in renal tubular cells [11,12,13]. Studies in the kidneys have shown that miR-29 can affect the expression of VEGFA, COL4A1, and COL4A2 by regulating the PI3K/AKT signaling pathway [14]. Genome-wide gene expression and in silico database analyses shows that miR-29 is also important in the regulation of renal cell carcinoma [15].

To investigate the regulatory effect of hypoxia on the extracellular matrix in renal cancer, we analyzed the sequencing results of hypoxia-treated renal cancer. The analysis of miRNAs showed that the expression of miR-29b under hypoxic conditions was only 16% of that under normoxia. Given the important implications of collagen IV for normal renal tissue, as well as tumors, we investigated the regulatory role of miR-29 for collagen IV. The sequences that are complementary to the miR-29 seed sequence exist in the 3′UTRs of *Col4a1*–*Col4a6* in humans, mice, and multiple species. To verify the regulatory sites of miR-29 on the collagen IV subunit, the corresponding sites of the 3’UTRs of *Col4a3*–*Col4a6* were cloned into a psiCHECK^TM^-2 vector, and the vectors with the corresponding mutated sites, were also constructed. The results showed that miR-29 could act on a conserved site (7186–7192) and two poorly conserved sites (7210–7216 and 8256–8262) of the *Col4a3* gene. For *Col4a4* and *Col4a6*, miR-29 could act on their complementary conserved sites at 5489–5495 and 5530–5536, respectively. Two conserved sites and one poorly conserved site of *Col4a5* were also regulated by miR-29. The study has theoretical implications for the use of miR-29 for the treatment of fibrosis, tumorigenesis and metastasis. 

## 2. Materials and Methods

### 2.1. Data Sources for Differentially Expressed miRNAs under Hypoxic Conditions

In previous work, mouse renal tumor cell lines (Renca cells) were used to perform hypoxia stress and sequencing [16]. The culture conditions were 21% pO*_2_* and 5% CO*_2_* for the control group (standard cell culture incubator), 1% pO*_2_* and 5% CO*_2_* for the experimental group (XVivo X3 workstation). Cells were harvested after 72 h of culture under hypoxic conditions, and sequencing was performed with the NextSeq 500 system. The sequencing data were submitted to the GEO database (GSE197301).

### 2.2. Target Gene Cluster Analysis

The target genes of miR-29 were analyzed using miRWalk (Mouse, miRWalk version 3, University of Heidelberg, Heidelberg, Germany) and TargetScan7.2 (Mouse, version 7.2, Whitehead Institute for Biomedical Research, Boston, MA, USA) (Supplementary Table S1). Jevnn [17] was used to obtain the intersection of target genes analyzed with miRWalk and TargetScan7.2, and the related genes in the intersection set were clustered and visualized by the clusterProfiler package of R project. Genome-wide annotation for mouse used org.Mm.eg.db in the OrgDb package in Bioconductor.

### 2.3. Sequence Analysis of 3’UTRs of Type IV Collagen Subunits

The 3’UTRs sequences of collagen IV subunits in human, chimp, rhesus, cow, dog, rat, mouse, opossum, and chicken were downloaded from the GenBank database (Supplementary Data S1). Sequence analyses (multiple sequence alignment and distance matrix calculation) were performed, and phylogenetic trees were constructed with MEGA 11 (Neighbor joining). The seed sequence and flanking sequences (Supplementary Data S2) were analyzed and displayed using WebLogo (version 3, University of California, Berkeley, CA, USA) [18].

### 2.4. Vector Construction

The 3’UTRs (including the putative miR-29 binding sites) of *Col4a3*–*Col4a6* were amplified via PCR using mouse (C57BL/6) cDNA as template, and the corresponding sequence was cloned into the 3’UTR of Renilla luciferase of the psiCHECK^TM^-2 vector. The miR-29 binding sites on the corresponding sequence were mutated with fusion PCR and cloned into the 3’UTR of Renilla luciferase.

### 2.5. Luciferase Assay

The psiCHECK^TM^-2 (Promega (Beijing) Biotech Co., Beijing, China) vector contains Firefly luciferase and Renilla luciferase. There is a multiple cloning site in the 3′UTR of Renilla luciferase. Renilla luciferase expression is affected when the sequence ligated into the multiple cloning site is regulated. With Firefly luciferase as an internal control, the regulatory effect of miRNA on the corresponding sequence can be detected.

MiR-29a or miR-29b mimics (Guangzhou RiboBio Co., Ltd., Guangzhou, China), as well as the corresponding psiCHECK™-2 vector, were co-transfected into HEK293T cells. HEK293T cells were previously preserved in our laboratory. The medium used for culturing HEK293T cells was DMEM that contained 10% FBS. The CO_2_ concentration of the incubator was 5%.

The number of cells seeded was 0.8 × 10^5^ (1 well of 24-well plate). Transfection was performed 12 h after cell seeding with Lipo6000™ Transfection Reagent (Beyotime Biotech. Inc., Shanghai, China). The activities of firefly luciferase and Renilla luciferase were detected 24 h after transfection.

### 2.6. Statistical Analysis

A Venn diagram was generated with jvenn [17]. Statistical analyses were performed using SPSS 16.0 (IBM, Armonk, NY, USA). The experimental results were presented as the mean ± SEM and were analyzed using Student’s *t*-test. Statistically significance at *p* < 0.05 is indicated by *, and statistical significance at *p* < 0.01 is indicated by **. The inhibition efficiency of miR-29 on the corresponding site is defined as the ratio of the effect of miR-29 on the relative luciferase activity after the site mutation minus the ratio of the effect of miR-29 on the relative luciferase activity before the site mutation.

## 3. Results

**Figure 1:**
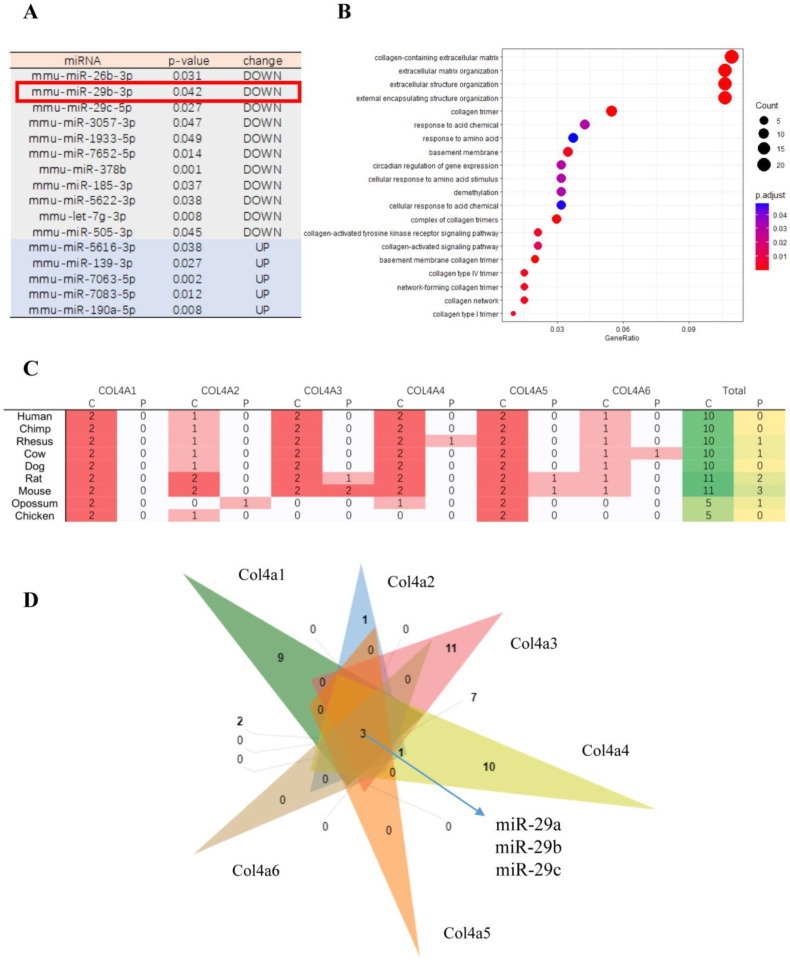
MiR-29 target genes analysis. (**A**) Significantly differentially expressed miRNAs after hypoxia treatment (*p* < 0.05). (**B**) Top 20 significantly enriched GO functional categories. (**C**) Statistics on the number of binding sites in collagen IV subunits of different species. C: conserved site; P: poorly conserved site. (**D**) Venn diagram showing the overlapping miRNA among the miRNAs regulating *Col4a1* to *Col4a6* for mouse.

We first analyzed the published sequencing results of hypoxia-treated renal cancer cell lines (murine Renca) [16]. The expressions of 16 miRNAs changed significantly (*p* < 0.05), including miR-29b (Figure 1A). MiR-29 is closely associated with the extracellular matrix. Thus, hypoxia can affect the expression of extracellular matrix related genes by regulating miR-29. We next performed an analysis of the predicted target genes of miR-29. The target genes of miR-29a, b, c were screened using miRWalk and TargetScanMouse 7.2, and the intersection of these target genes was clustered according to gene functions by GO (Gene Ontology). Figure 1B shows the top 20 terms. According to these results, the predicted miR-29 target genes were most closely related to the extracellular matrix, collagen, and the basement membrane.

Collagen IV is important for normal renal tissue, as well as for renal cancer. For several species, such as mice and humans, *Col4a1*–*Col4a6* all contain complementary sites with miR-29 seed sequences (Figure 1C,D).

**Figure 2:**
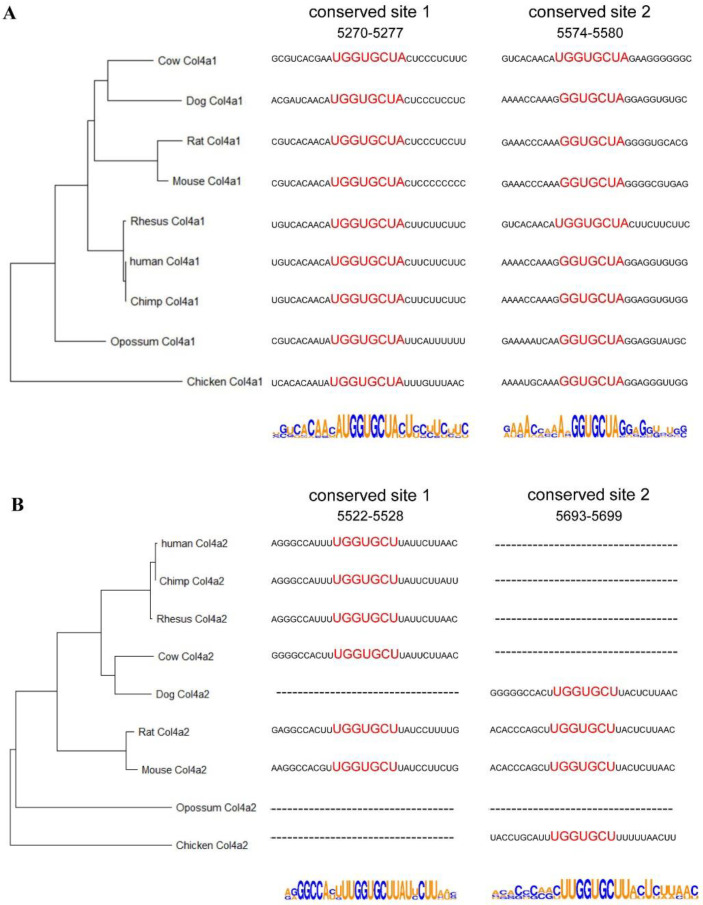
Analysis of *Col4a1* and *Col4a2* 3’UTRs and miR-29 binding sites. (**A**,**B**) Phylogenetic tree constructed from the 3’UTRs of *Col4a1* and *Col4a2* in multiple species. Analysis of miR-29 binding sites and flanking sequences in the 3’UTRs of *Col4a1* and *Col4a2* in multiple species.

Therefore, we analyzed and examined whether miR-29 could regulate the expression of collagen IV through the predicted binding site. First, we analyzed the 3’UTRs of *Col4a1* and *Col4a2*. Based on the 3’UTR sequences of *Col4a1* and *Col4a2*, we analyzed the similarities of the sequences of multiple species and constructed phylogenetic trees (Figure 2A,B). The phylogenetic tree constructed with the 3’UTRs of *Col4a1* was not consistent with the morphological classification.

We also analyzed the miR-29 binding sites and flanking sequences in the 3’UTRs of *Col4a1* and *Col4a2* in multiple species (Figure 2A,B). In mice, both *Col4a1* and *Col4a2* contained two miR-29 binding sites in the 3’UTRs. For the *Col4a1* gene, there were corresponding sequences in the eight species analyzed in present work. Meanwhile for the *Col4a2* gene, the sequence from 5522 to 5528 did not exist in dogs, opossums and chickens, while the sequence from 5693 to 5699 did not exist in humans, chimpanzees, rhesus monkeys, cattle and opossums. The regulatory role of miR-29 on the corresponding sequences of *Col4a1* and *Col4a2* have been studied [19]. The regulation of *Col4a1* by miR-29a and miR-29b is mainly controlled through the sequence from 5270 to 5276. MiR-29a and miR-29b regulates *Col4a2* mainly through the sequences 5693–5699, and 5522–5528 [19].

**Figure 3:**
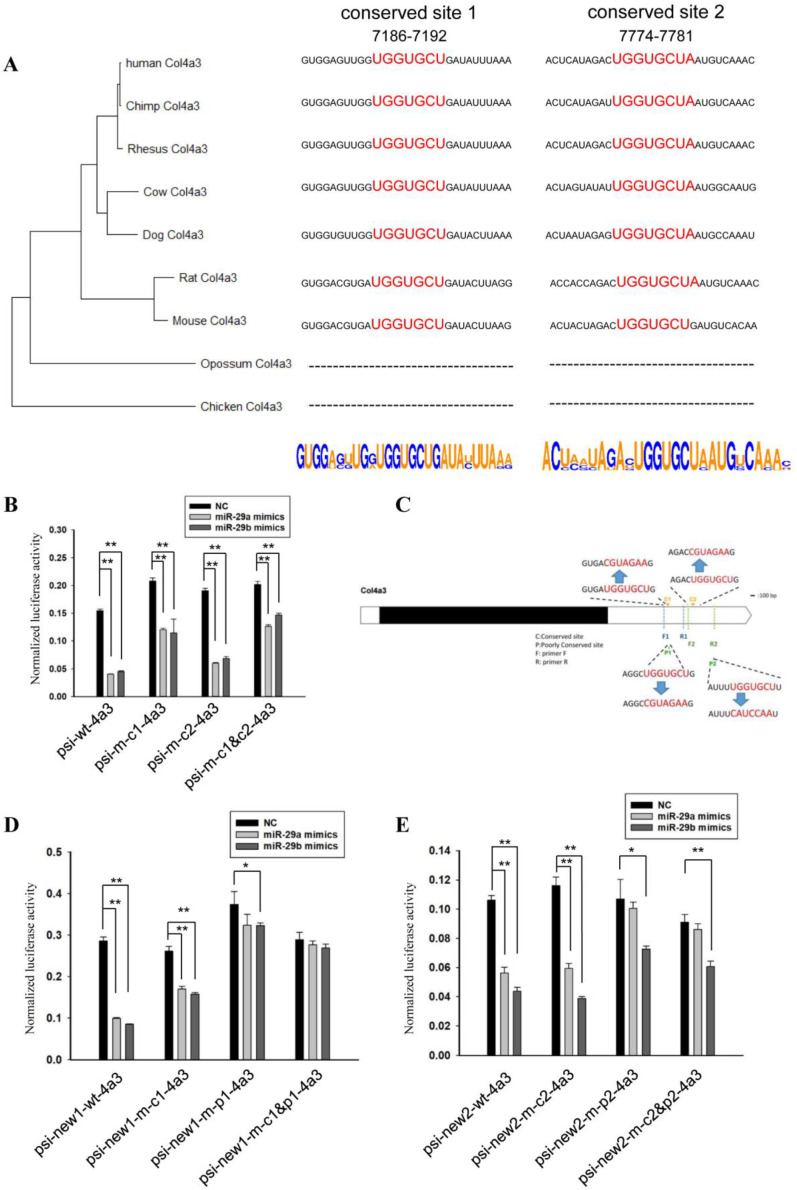
MiR-29 directly regulates the 3’UTR of *Col4a3*. (**A**) Phylogenetic tree constructed from the 3’UTRs of *Col4a3* in multiple species. Analysis of miR-29 binding sites and flanking sequences in the 3’UTRs of *Col4a3* in multiple species. (**B**) The effect of miR-29a or miR-29b mimics on the relative luciferase activity of the psi-wt-4a3 vector (psiCHECK^TM^-2 containing wild-type 3’UTR of *Col4a3*), psi-m-c1-4a3 vector (psiCHECK^TM^-2 contained 3’UTR of *Col4a3* mutated at conserved site 1), psi-m-c2-4a3 vector, and the psi-m-c1&c2-4a3 vector (psiCHECK^TM^-2 containing 3’UTR of *Col4a3* mutated at conserved sites 1 and 2) was examined. The data are shown as the mean ± SEM (*n* = 3). **, *p* < 0.01. (**C**) Schematic diagram of complementary sequence sites and primer F1, R1, F2, and R2 sites. (**D**) The effect of miR-29a or miR-29b mimics on the relative luciferase activity of the psi-new1-wt-4a3 vector, psi-new1-m-c1-4a3 vector, psi-new1-m-p1-4a3 vector, and the psi- new1-m-c1&p1-4a3 vector was examined. The data are shown as the mean ± SEM (*n* = 3). *, *p* < 0.05; **, *p* < 0.01. (**E**) The effect of miR-29a or miR-29b mimics on the relative luciferase activity of the psi-new2-wt-4a3 vector, psi-new2-m-c2-4a3 vector, psi-new2-m-p2-4a3 vector, and the psi-new2-m-c2&p2-4a3 vector was examined. The data are shown as the mean ± SEM (*n* = 3). *, *p* < 0.05; **, *p* < 0.01.

Next, we analyzed the regulatory effect of miR-29 on the 3’UTRs of *Col4a3*. For mouse, the *Col4a3* included two conserved and two poorly conserved regions that are complementary to the miR-29 seed sequence. A phylogenetic tree constructed by the 3’UTRs of *Col4a3* in different species was consistent with the morphological classification (Figure 3A). The poorly conserved sequence 7210–7216 of the mouse *Col4a3* gene only existed in mice, and the poorly conserved sequence 8262–8268 only existed in mice and rats; thus, only the conserved regions complementary to the miR-29 and flanking sequences were analyzed (Figure 3A).

In order to analyze the regulatory effect of miR-29 on the 3’UTR of *Col4a3*, we designed primers based on the mouse sequence; the amplified product contained two conserved binding sites of miR-29 and a poorly conserved site between them. At the same time, we mutated the two conserved binding sites of miR-29 (separately or simultaneously) and ligated the normal sequence as well as the mutated sequences into the 3’UTR of Renilla luciferase of the psiCHECK^TM^-2 vector.

Vectors containing the wild-type sequence (psi-wt-4a3), sequences mutated at conserved site 1 (psi-m-c1-4a3), mutated conserved site 2 (psi-m-c2-4a3) or mutated at both sites (psi-m-c1&c2-4a3) were co-transfected with miR-29a mimics, miR-29b mimics or controls, respectively. At 24 h after transfection, the relative activity of Renilla luciferase was analyzed using firefly luciferase as an internal reference; the results ae shown in Figure 3B. Relative luciferase activity was reduced when the psi-wt-4a3 vector was co-transfected with miR-29a or miR-29b mimics. When conserved site 1 was mutated, the inhibition of miR-29 on relative luciferase activity decreased. However, the mutation of conserved site 2 did not affect the inhibition of relative luciferase activity by miR-29. Consistently, when conserved site 1 and site 2 were all mutated, miR-29 still had a regulatory effect on this sequence, which was similar to the effect of mutating site 1 alone. It could be seen that miR-29 had no regulatory effect on the conserved site 2, and miR-29 may play a regulatory role through the poorly conserved site.

Considering the importance of the poorly conserved site, we re-amplified this sequence. As shown in Figure 3C, the fragment amplified by primers F1 and R1 contained a conserved site and a poorly conserved site. The fragment amplified by primers F2 and R2 contained another conserved site and a poorly conserved site. We also mutated the corresponding sites and ligated the wild-type sequences or the mutated sequence into the psiCHECK^TM^-2 vector. Vectors that contained the wild-type sequence or sequences mutated at binding sites were co-transfected with miR-29a mimics, miR-29b mimics or controls.

Consistent with the results in Figure 3C, when conserved site 1 was mutated, the effect of miR-29 on relative luciferase activity decreased, but the range was limited. However, when the poorly conserved site 1 was mutated, the effect of miR-29 on relative luciferase activity greatly decreased. When both conserved site 1 and poorly conserved site 1 were mutated, miR-29 no longer affected relative luciferase activity (Figure 3D).

Corresponding experimental results for another fragment showed that mutation of conserved site 2 did not affect the function of miR-29a on relative luciferase activity. When the poorly conserved site 2 was mutated, there was no significant difference between transfected miR-29a and the control (Figure 3E). In the results of this experiment, the effects of miR-29a and miR-29b on the same vector were clearly different. It is speculated that there may be special sites in these sequences which miR-29b can bind and miR-29a cannot.

**Figure 4:**
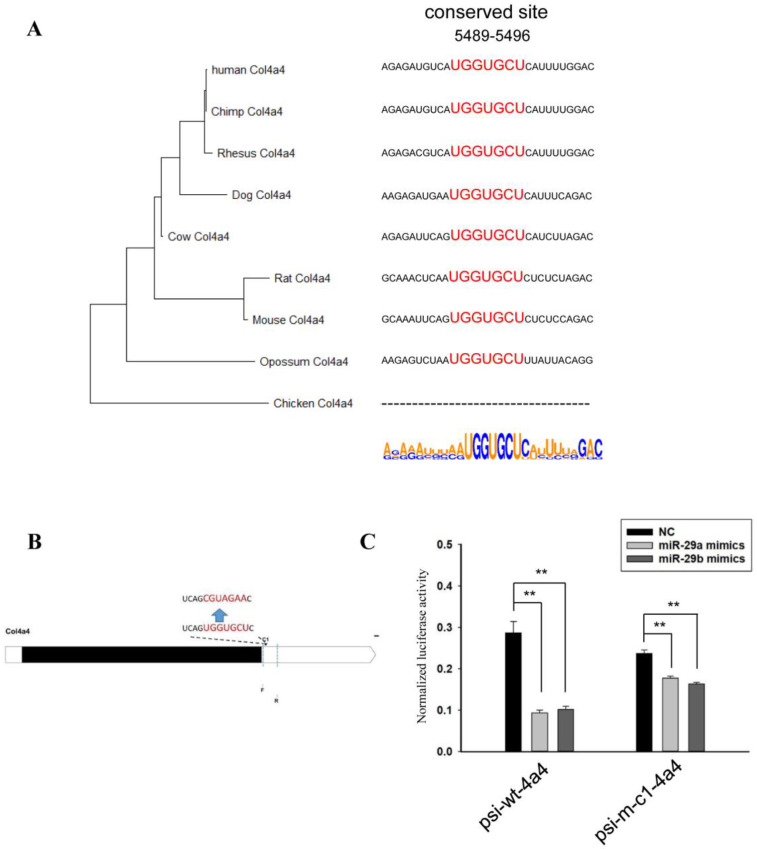
MiR-29 directly regulated the 3’UTR of *Col4a4*. (**A**) Phylogenetic tree constructed from the 3’UTRs of *Col4a4* in multiple species. Analysis of miR-29 binding sites and flanking sequences in the 3’UTRs of *Col4a3* in multiple species. (**B**) Schematic diagram of complementary sequence site and primer sites. (**C**) The effect of miR-29a or miR-29b mimics on the relative luciferase activity of the psi-wt-4a4 vector and psi-m-c1-4a4 vector was examined. The data are shown as the mean ± SEM (*n* = 3). **, *p* < 0.01.

Next, we analyzed the effect of miR-29 on *Col4a4*. First, a phylogenetic tree was constructed based on the sequences of the 3’UTRs of *Col4a4*. The results of the phylogenetic tree were not completely consistent with the morphological classification (Figure 4A), with the main differences observed in the results of dog and cow. The sequences of the binding site and the flanking sequences were analyzed (Figure 4A).

As shown in Figure 4B, we amplified and mutated the corresponding sequences and ligated the corresponding sequences into the psiCHECK^TM^-2 vector. The results of the co-transfection of the mimics and the vector are shown in Figure 4C. MiR-29 significantly reduced the relative activity of luciferase, but when the corresponding site was mutated, miR-29a and miR-29b still had certain effects. Except for the known site in this sequence, there was no other site that contained multiple consecutive nucleotides complementary to miR-29. Therefore, miR-29 may indirectly affect the relative activity of luciferase through other genes.

**Figure 5:**
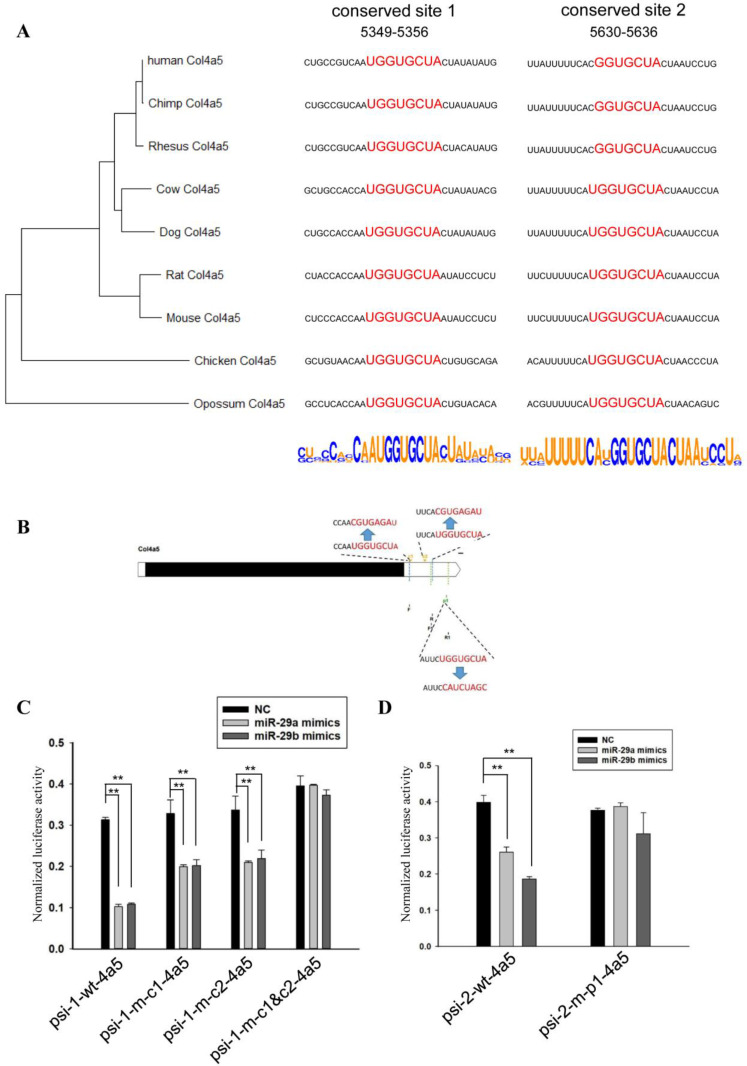
MiR-29 directly regulates the 3’UTR of *Col4a5*. (**A**) Phylogenetic tree constructed from the 3’UTRs of *Col4a5* in multiple species. Analysis of miR-29 binding sites and flanking sequences in the 3’UTRs of *Col4a5* in multiple species. (**B**) Schematic diagram of complementary sequence sites and primer sites. (**C**) The effect of miR-29a or miR-29b mimics on the relative luciferase activity of the psi-1-wt-4a5 vector, psi-1-m-c1-4a5 vector, psi-1-m-c2-4a5 vector, and the psi-1-m-c1&c2-4a5 vector was examined. The data are shown as the mean ± SEM (*n* = 3). **, *p* < 0.01. (**D**) The effect of miR-29a or miR-29b mimics on the relative luciferase activity of the psi-2-wt-4a5 vector and psi-2-m-p1-4a5 vector was examined. The data are shown as the mean ± SEM (*n* = 3). **, *p* < 0.01.

For the *Col4a5* gene, we first established a phylogenetic tree based on the 3’UTRs (Figure 5A). The *Col4a5* gene contained two conserved sites and one poorly conserved site that could be complementary to miR-29. In the species we analyzed, the poorly conserved site was found only in mice and rats. Therefore, we showed the two conserved sites and the flanking sequences (Figure 5A).

The three sites were divided into two fragments for study, where fragment 1 contained two conserved sites and fragment 2 contained the remaining poorly conserved site (Figure 5B). We amplified and mutated the sequences and ligated them into the psiCHECK^TM^-2 vector. Using miR-29 mimics and vectors to analyze the regulation sites of miR-29, it was found that miR-29 had a strong inhibitory effect on wild-type fragment 1 (Figure 5C). When the two conserved sites were mutated separately, the inhibition ratios were reduced. When both sites were mutated, miR-29 no longer affected the relative activity of luciferase.

For the poorly conserved site, taking miR-29a as an example, when the vector containing the poorly conserved site was co-transfected with miR-29a mimics, the relative activity decreased (Figure 5D). After the corresponding site mutation, co-transfection of miR-29a and the vector no longer affected the relative activity of luciferase.

**Figure 6:**
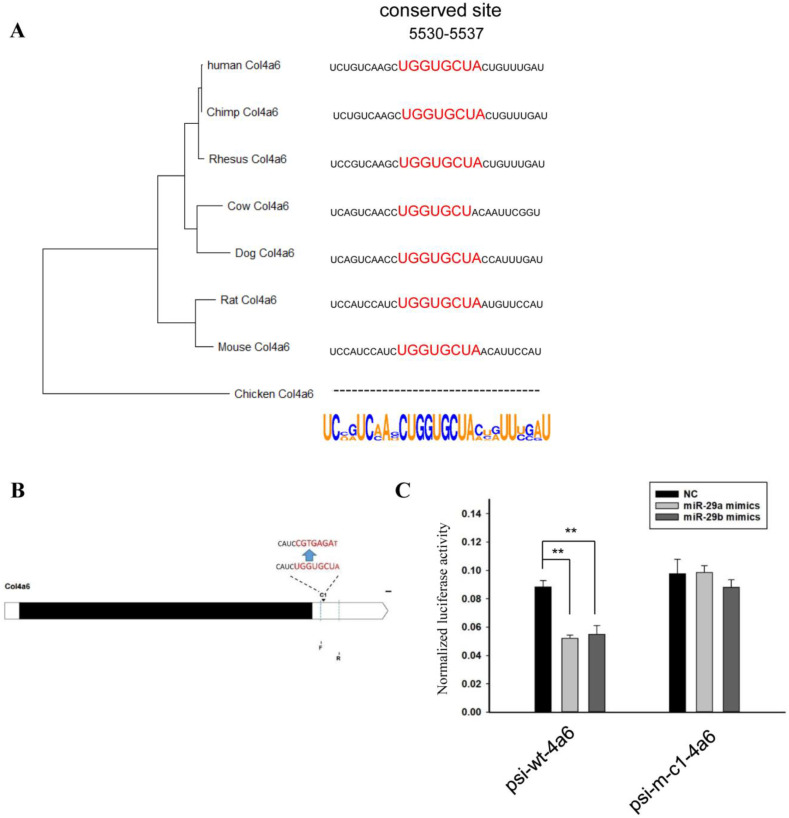
MiR-29 directly regulates the 3’UTR of *Col4a6*. (**A**) Phylogenetic tree constructed from the 3’UTRs of *Col4a6* in multiple species. Analysis of miR-29 binding site and flanking sequences in the 3’UTRs of *Col4a6* in multiple species. (**B**) Schematic diagram of complementary sequence site and primer sites. (**C**) The effect of miR-29a or miR-29b mimics on the relative luciferase activity of the psi-wt-4a6 vector and the psi-m-c1-4a6 vector was examined. The data are shown as the mean ± SEM (*n* = 3). **, *p* < 0.01.

Finally, we analyzed the 3’UTRs of *Col4a6*. A phylogenetic tree was established based on the 3’UTRs, which was consistent with morphological analysis (Figure 6A). Then the flanking sequences of this conserved site complementary to miR-29 on 3’UTR were analyzed (Figure 6A). The corresponding sequence was amplified, mutated and ligated into the psiCHECK^TM^-2 vector (Figure 6B). The vector and miR-29 were co-transfected into HEK293T cells. The relative activity of luciferase detection results showed that miR-29 had an inhibitory effect on this fragment (Figure 6C). When the corresponding site was mutated, miR-29 no longer had an inhibitory effect on this fragment.

## 4. Discussion

Hypoxia induces changes in the extracellular matrix of tumor cells [20], and collagen IV is an important component of the extracellular matrix [21]. In this study, we analyzed the sequencing results of renal cancer cells under hypoxia and found that the expression of miR-29b was changed. Collagen IV is the predicted target gene of miR-29. We also analyzed the sequences of the 3’UTRs of different subunits of collagen IV in different species and constructed phylogenetic trees. Moreover, we examined the inhibitory effect of miR-29 on multiple sites existing in mouse *Col4a3*–*Col4a6*. These results could provide a reference for study of the regulatory mechanism and function of hypoxia and collagen IV.

Genes are subject to multiple regulation in the process of transcription and translation, among which miRNAs play an important role in gene regulation by 3’UTR [22]. For a particular gene, the selection pressure during its evolution may even come mainly from 3’UTR [23]. Therefore, we analyzed variations in the 3’UTRs of the collagen IV subunit in different species. For *Col4a2*, *Col4a3*, *Col4a5*, and *Col4a6*, the phylogenetic trees that were established based on the 3’UTRs were consistent with the morphological classification. However, this was not the case with *Col4a1* and *Col4a4*. It may be that in different species, *Col4a1* and *Col4a4* have special functions, and that 3’UTRs are regulated differently. It is also possible that the 3’UTRs of these two genes were not screened by positive selection. Subsequent studies can be carried out to investigate whether *Col4a1* and *Col4a4* have specific functions in different species.

MiR-29 can inhibit the expression of various fibrosis-related proteins through 3’UTR [24]. Our analysis of mouse *Col4a1*–*Col4a6* showed that these six subunits all had miR-29 binding sites in the 3’UTR, and *Col4a1*, *Col4a2*, *Col4a3*, and *Col4a5* contained multiple binding sites. MiRNAs inhibit gene expression through fine regulation. MiRNAs can enhance their inhibitory effects by acting on multiple sites of the same gene, as well as acting on multiple subunits or multiple genes in a certain signaling pathway [25]. Therefore, miR-29 can enhance the inhibition of the extracellular matrix through multiple sites and multiple genes.

In this study, we detected eight sites on the four genes, *Col4a3*–*Col4a6*, of which five sites were conserved across multiple species and three sites were poorly conserved. MiR-29 had important regulatory effects for all three poorly conserved sites. One of the five conserved sites was not regulated by miR-29. From the above results, it was found that poorly conserved sites were also very important.

In multiple experimental results, the roles of miR-29a and miR-29b were observed to not exactly be the same. Especially after the predicted binding site mutation, miR-29a usually no longer had an inhibitory effect, while miR-29b still had some inhibitory effect (Figure 3E, Figure 5C,D, Figure 6C). MiRNAs can play a regulatory role through non-canonical sites [26,27,28], and miR-29b may act on other sites of the vector.

The analysis of the 3’UTRs of collagen IV subunits and the study on the regulation of specific sites on the 3’UTRs by miR-29 could provide reference for the study of hypoxia and the extracellular matrix.

## Data Availability

miRNA microarray of hypoxia-treated renal cancer cell lines (GSE197301).

## Supplementary Materials

The following supporting information can be downloaded at: https://www.mdpi.com/article/10.3390/biomedicines10123286/s1, Supplementary Data S1: Supplementary Data S1; Supplementary Data S2: Supplementary Data S2; Table S1: Supplementary Table S1e.
